# Harnessing helminth-driven immunoregulation in the search for novel therapeutic modalities

**DOI:** 10.1371/journal.ppat.1008508

**Published:** 2020-05-14

**Authors:** Stephanie M. Ryan, Ramon M. Eichenberger, Roland Ruscher, Paul R. Giacomin, Alex Loukas

**Affiliations:** Centre for Molecular Therapeutics, Australian Institute of Tropical Health and Medicine, James Cook University, Cairns, Queensland, Australia; University of Pennsylvania, UNITED STATES

## Abstract

Parasitic helminths have coevolved with humans over millennia, intricately refining and developing an array of mechanisms to suppress or skew the host’s immune system, thereby promoting their long-term survival. Some helminths, such as hookworms, cause little to no overt pathology when present in modest numbers and may even confer benefits to their human host. To exploit this evolutionary phenomenon, clinical trials of human helminth infection have been established and assessed for safety and efficacy for a range of immune dysfunction diseases and have yielded mixed outcomes. Studies of live helminth therapy in mice and larger animals have convincingly shown that helminths and their excretory/secretory products possess anti-inflammatory drug-like properties and represent an untapped pharmacopeia. These anti-inflammatory moieties include extracellular vesicles, proteins, glycans, post-translational modifications, and various metabolites. Although the concept of helminth-inspired therapies holds promise, it also presents a challenge to the drug development community, which is generally unfamiliar with foreign biologics that do not behave like antibodies. Identification and characterization of helminth molecules and vesicles and the molecular pathways they target in the host present a unique opportunity to develop tailored drugs inspired by nature that are efficacious, safe, and have minimal immunogenicity. Even so, much work remains to mine and assess this out-of-the-box therapeutic modality. Industry-based organizations need to consider long-haul investments aimed at unraveling and exploiting unique and differentiated mechanisms of action as opposed to toe-dipping entries with an eye on rapid and profitable turnarounds.

## Introduction

Parasitic worms (helminths) infect approximately 2 billion people worldwide, predominantly children in rural subtropical and tropical areas with inadequate sanitation [1]. Helminths have struck a balance with their hosts, refined by millennia of coevolution, to meet their needs for propagation and transmission while minimizing pathology [2]. They promote wound healing and tissue repair and skew distinct immune processes to improve their long-term survival. Arguably, the most masterful trait of parasitic helminths, from a drug development perspective, is their ability to potently regulate host inflammatory responses.

## Helminth-mediated prevention of inflammatory and metabolic disorders in people

In industrialized nations, there has been a reduction in exposure to infectious agents because of vaccination, increased sanitation, improved hygienic standards, and widespread use of antibiotics. The vast decline in the prevalence of contagious diseases from these communities, helminthiases, in particular, is inversely associated with an alarming increase in the incidence of inflammatory and metabolic disorders [3]. For example, the Western world is experiencing an increasing rate of inflammatory bowel disease (IBD), and at present, there is no available cure. Compounding matters, there has been a sharp rise in the incidence of IBD and allergies in the newly industrialized nations of Asia and Latin America [4]. This increase in noncommunicable diseases is, at least in part, a result of diets becoming westernized, decreased exposure to infections, large scale deworming programs, and mass migration [5].

Although the association between helminths and inflammatory diseases is multifactorial, selective pressure placed on the human genome by historically widespread helminth infection has driven various polymorphisms, including at loci associated with predispositions to inflammatory diseases [6]. Moreover, minimal exposure to pathogens results in an underdeveloped regulatory immune network, culminating in an increased prevalence of disorders that result from immune dysfunction [7, 8]. Helminth-mediated protection is not, however, restricted to autoimmune and allergic diseases. There is an inverse relationship observed between human helminth infection, insulin resistance, and type 2 diabetes (T2D) [9, 10]. It has been proposed that chronic helminth infection results in long-term beneficial effects on host metabolism, especially on white adipose tissue (WAT), intestines, and liver [11]. Understanding the molecular mechanisms of WAT inflammation is topical in drug development given the epidemic of metabolic diseases, and we will touch upon this again later in the review.

**Fig 1:**
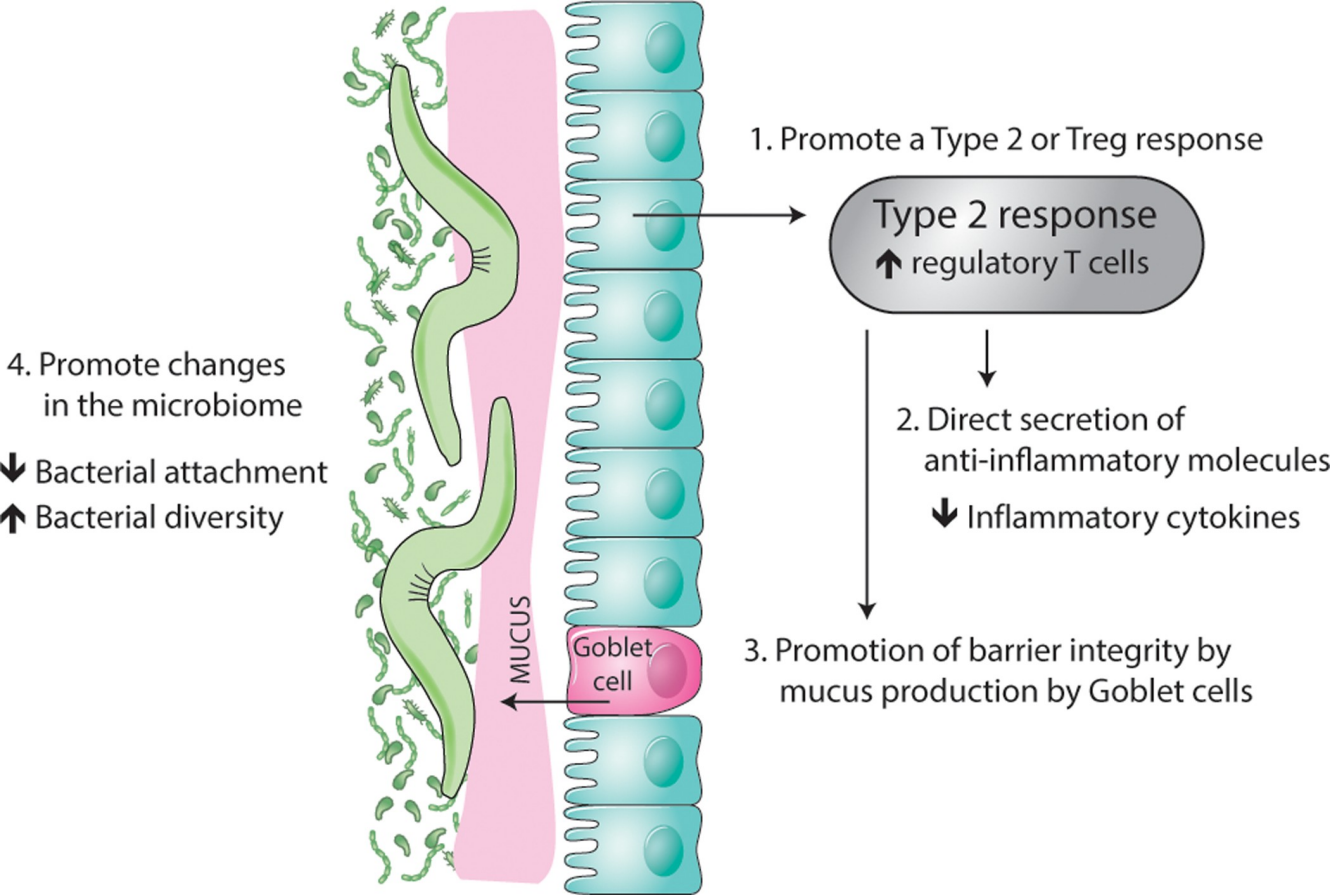
"Worm therapy" for immune dysregulation diseases. The range of host physiological factors impacted by gastrointestinal helminth infection could alleviate inflammatory disease. (1) Parasite-derived factors drive an inclusive or exclusive polarized regulatory or type 2 response, which is responsible for (2) direct secretion of anti-inflammatory molecules from the host immune system (3) and the promotion of barrier integrity, which is often compromised in the pathophysiology of IBD and foodborne incompatibilities. Furthermore, (4) helminth colonization provides factors for a diverse bacterial environment that protects against gut inflammation. IBD, inflammatory bowel disease; T_*H*_2, T helper type 2; Treg, regulatory T cell.

The mechanisms by which parasitic helminths regulate inflammation and metabolism are diverse and complex and have been reviewed extensively [11–15]. Helminths are potent drivers of T helper type 2 (T_H_2) immune responses, characterized by eosinophilia, mast cell mastocytosis, type 2 innate lymphoid cells (ILC2s), tuft cells, and mucus production. Overlaid on this T_H_2 response, however, is a predominant state of immune tolerance, characterized by an abundance of IL-10 produced by regulatory cell populations such as regulatory T cells (Tregs), regulatory B cells (Bregs), tolerogenic dendritic cells (DCs), and alternatively activated macrophages. Furthermore, the interactions between helminths, the microbiome and its attendant metabolites [16–21], and the nervous system have become increasingly important (Fig 1).

## Clinical trials of experimental human helminth infections for treating inflammatory diseases

Experimental helminth infections have been used in the treatment of allergic and autoimmune diseases in human clinical trials for over 15 years and have yielded promising results (Table 1). Two helminth species have been used in clinical trials for treating inflammatory diseases—the pig whipworm *Trichuris suis* and the human hookworm *Necator americanus*. The therapeutic potential of orally administered *T*. *suis* ova (TSO) has been assessed in phase 1 trials in patients with the 2 primary forms of IBD—Crohn’s disease and ulcerative colitis. After 12 weeks of therapy, significant improvement according to the intent-to-treat principle occurred in patients receiving TSO compared with those who received placebo [22]. TSO was also assessed in an open-label clinical trial in Crohn’s disease patients, in which 72% of subjects were in remission after 24 weeks [23]. Disappointingly, although early trials with *T*. *suis* showed promise, subsequent phase 2 trials failed to reach their clinical endpoints in both IBD [24] and multiple sclerosis [25, 26].

**Table 1:** Completed and ongoing therapeutic clinical trials using helminth products in humans in disease settings. These trials are rigorously assessing the safety and tolerability of experimental helminth infection and therapeutic efficacy of infections in disease indications.

Trial/phase	Helminth	Status (year range)	Study title and treatment	Results outcome	Reference
**Phase I**	TSO	2003 (complete)	**Crohn’s disease** Initial safety studies of oral inoculation (2,500 ova) over 12 weeks (*n* = 7).	Patients displayed clinical improvements and no serious adverse events.	[128]
**NCT01433471** **Phase 1 and 2**	TSO	2005 (complete)	**Ulcerative colitis** Open-label study randomized, double-blind, placebo-controlled oral inoculation (2,500 ova) over 12 weeks (*n* = 30).	Improvement in disease index by 43% in treatment cohort.	[23]
**EUCTR2011-006344-71-DE** **Phase 1**	TSO	2008 to 2011 (terminated)	**Rheumatoid arthritis** Oral inoculation (2,500 ova) in 2-week intervals for 24 weeks (*n =* 50).	Trial terminated and results unknown.	Immanuel Hospital Berlin, Germany
**NCT00645749** **Phase 2**	TSO	2008 to 2015 (complete)	**Multiple sclerosis** (HINT) Oral inoculation (2,500 ova) 2-week intervals for 12 weeks (*n =* 17).	Trend toward 35% diminution in active lesions. Increase in T regulatory lymphocytes with treatment. Increase in serum levels of IL-4 and IL-10 during treatment. No serious adverse events.	[129]
**NCT01006941** **Phase 2**	TSO	2009 to 2011 (complete)	**Multiple sclerosis** Nonrandomized, open-labeled oral inoculation (2,500 ova) 2-week intervals for 12 weeks (*n =* 10).	No obvious benefit observed in infection group. Mild to self-limiting adverse events.	[25]
**EudraCT no. 2007-006099-12** **Phase I**	TSO	2007 to 2010 (complete)	**Allergic rhinitis** Randomized, double-blind, placebo-controlled, 2,500 ova administered (*n =* 49) and placebo (*n =* 47).	No therapeutic effect on allergic rhinitis of infection.	[130]
**NCT01413243** **Phase 2**	TSO	2011 to 2016 (terminated)	**Multiple sclerosis** (TRIOMS) randomized control trial of oral inoculation (2,500 ova) 2-week intervals for 12 weeks (*n* = 50).	Unknown	[131]
**NCT01434693** **Phase 1**	TSO	2011 to 2013 (complete)	**Crohn’s disease** Randomized, double-blind, placebo-controlled sequential oral dose escalation (500, 2,500, 7,500 ova) (*n* = 36).	Placebo and treatment groups experience minor adverse events. No obvious improvement in pathology with infection.	[132]
**NCT01576471** **Phase 2**	TSO	2013 (unknown)	**Crohn’s disease** (TRUST-1) Randomized, double-blind, placebo-controlled oral inoculation of 7,500 ova in 2-week intervals. Placebo group included.	Unknown results of study	Coronado Biosciences, United Kingdom
**NCT01279577** **Phase 2**	TSO	2011 to 2015 (complete)	**Crohn’s disease** Randomized, double-blind, placebo-controlled, low, medium, high oral inoculation ova (*n* = 254) participants.	Unknown results of study	Dr. Falk Pharma, Germany
**NCT01836939** **Phase 1**	TSO	2013 to 2015 (complete)	**Plaque psoriasis** Randomized, 2-arm trial of oral inoculation (2,500 ova) in 2-week intervals for 10 weeks and (7,500 ova) in 2-week intervals for 10 weeks (*n =* 8).	Unknown results of study	Icahn School of Medicine at Mount Sinai, US
**NCT01948271** **Phase 1**	TSO	2013 to 2016 (terminated)	**Plaque psoriasis** Open-label, oral inoculation (7,500 ova) in 2-week intervals for 14-week duration (*n* = 3).	Trial terminated because of a lack of efficacy	Tufts Medical Center, US
**NCT02011269** **Phase 2**	TSO	2013 to 2016 (withdrawn)	**Plaque psoriasis** Randomized, blinded, placebo-controlled, 3-arm trial of oral inoculation (7,500 ova) in 2-week intervals for 10 weeks, (15,000 ova) in 2-week intervals for 10 weeks.	Trial withdrawn and results unknown.	Coronado Biosciences, UK
**Phase 1**	*N*. *americanus* larvae	2006 (complete)	**Crohn’s disease** Proof of concept: inoculation with larvae at week 0 (*n* = 9) and week between week 27 to 30 (*n* = 5)	Remission at week 45 observed in 5 patients inoculated in week 0. No serious adverse events.	[31]
	*N*. *americanus* larvae	2009	**Allergic rhinoconjunctivitis** 30 individuals with allergic rhinoconjunctivitis were randomized and inoculated with 10 larvae or placebo and followed for 12 weeks.	Hookworm infection did not induce clinically significant exacerbation of airway responsiveness.	[133]
**NCT00469989**	*N*. *americanus* larvae	2010	**Asthma** Randomized, placebo-controlled, inoculation with 10 larvae and followed for 16 weeks (*n* = 30).	Hookworm infection did not significantly improve bronchial responsiveness nor other measures of asthma control.	[28]
**NCT00671138** **Phase 2**	*N*. *americanus* larvae	2007 to 2011 2011 to 2016 (completed)	**Celiac disease** Randomized, double-blinded, placebo-controlled trial. Part 1: inoculating celiac disease patients with the human hookworm *N*. *americanus* larvae at week 0 (*n* = 10) and week 12 (*n* = 10), followed by oral gluten challenge at week 20 of 16 g gluten per day for 5 days. Part 2: inoculating celiac disease patients with the *N*. *americanus* larvae at week 0 (*n =* 7) and week 12 (*n =* 7). At week 20, subjects were given an oral gluten challenge at week 20 of 16 g gluten per day for 5 days.	Infection conferred no obvious benefit to pathology. Mucosa of hookworm-infected subject maintained healthy appearance. No serious adverse events. Duodenal biopsy culture of hookworm-infected subjects had suppressed IL-17A and IFN-γ and increased levels of IL-10, IL-5 and regulatory T cells. No serious adverse events.	[30, 134]
**NCT01661933** **Phase 1 and 2**	*N*. *americanus* larvae	2012 to 2014 (completed)	**Celiac disease** Inoculating celiac disease patients with the human hookworm *N*. *americanus* larvae at week 0 (*n =* 10) and week 4 (*n =* 10), followed by incremental gluten challenge (*n =* 12).	Ten patients successfully tolerated gluten challenge. No serious adverse events.	[135]
**NCT02754609** **Phase I**	*N*. *americanus* larvae	2016 to 2020 (completed)	**Celiac disease** Hookworm therapy for celiac disease (NainCeD-3). Randomized, placebo (*n =* 10), inoculation week 0 and week 8 (*n =* 40) to assess safety and dose-ranging clinical trial examining sustained gluten challenge.	Manuscript in preparation	James Cook University, Australia
**NCT00630383** **Phase 2**	*N*. *americanus* larvae	2008 to 2012 (withdrawn)	**Multiple sclerosis** Randomized, inoculation 25 larvae at week 0. Placebo group included.	Withdrawn—superseded by a similar study	University of Nottingham, UK
**NCT01470521** **Phase 2**	*N*. *americanus* larvae	2011 to 2016 (complete)	**Multiple sclerosis** (WIRMS) Randomized, inoculation 25 larvae at week 0 (*n =* 36). Placebo group (*n =* 36).	Unknown study results	University of Nottingham, UK

HINT, Helminth-induced Immunomodulation Therapy; IFN-γ, interferon gamma; IL, interleukin; TRIOMS, *Trichuris Suis* Ova in Recurrent Remittent Multiple Sclerosis; TRUST-1, Treatment With Oral CNDO 201 *Trichuris Suis* Ova Suspension in Patients; TSO, *Trichuris suis* ova; WIRMS, Worms for Immune Regulation of Multiple Sclerosis.

**Fig 2:**
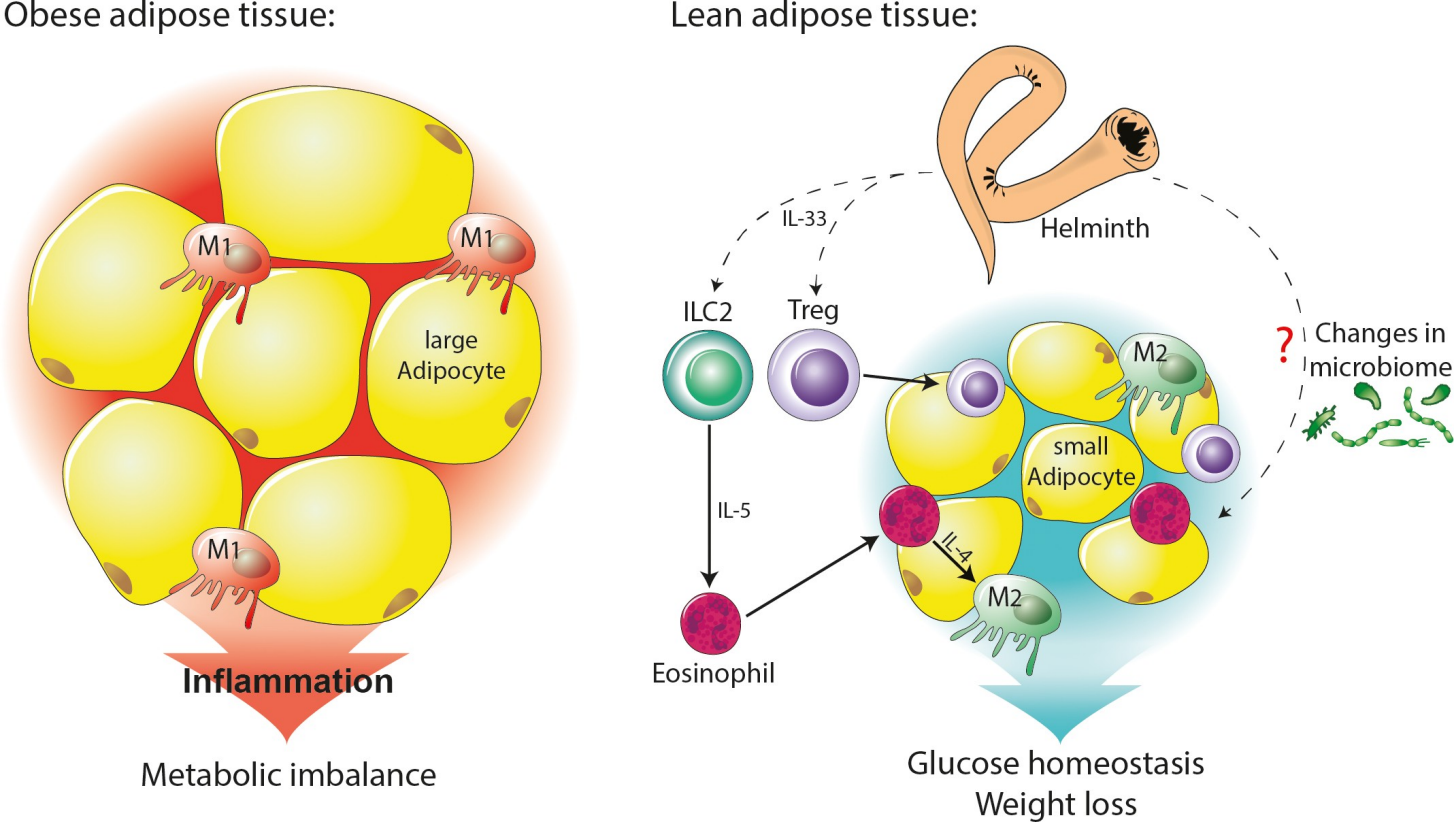
Inflammation and metabolic imbalance versus glucose homeostasis and weight loss, in response to infection with gastrointestinal nematodes and intravascular blood flukes and their ESPs. Chronic inflammation in adipose tissue is linked to a switch to M1 macrophages and the production of TNF-α and IL-1β. Helminth infection and helminth ESPs induce changes in the gut that lead to a regulatory/T_H_2 milieu that results in reduced inflammation in adipose tissue, enhanced glucose homeostasis, and decreased weight gain in obese animals. Furthermore, this regulatory/T_H_2 milieu increases IL-33 produced in adipose tissue by stromal cells within the progenitors of both adipocytes and mesenchymal cells. The production of IL-33 induces resident ILC2 to produce IL-5, which recruits eosinophils. Eosinophils in white adipose tissue secrete IL-4, which induces M2 macrophages. The production of IL-33 also induces regulatory T and B cells to produce IL-10, which sustains M2 macrophage activity. ESPs, excretory/secretory product; IL, interleukin; ILC2, type 2 innate lymphoid cell; M1, classically activated macrophage; M2, alternatively activated macrophage; T_H_2, T helper type 2; TNF, tumour necrosis factor.

*T*. *suis* establishes chronic infections in pigs but is expelled from the human body within weeks and therefore requires frequent dosing. On the other hand, *N*. *americanus* is primarily a human parasite and can survive for years in infected individuals [27]. Experimental infection with *N*. *americanus* is safe and well tolerated in human volunteers [28–30]. In a small phase 1 clinical trial for Crohn’s disease, percutaneous administration of *N*. *americanus* in relatively low doses was well tolerated, and patients who remained in the trial for 1 year were in disease remission [31]. A subsequent trial targeted celiac disease because of its histopathological similarities to IBD. Celiac subjects on a gluten-free diet who were experimentally infected with *N*. *americanus* displayed improved tolerance to escalating gluten micro-challenge, a decreased presence of inflammatory cytokine-producing T cells in the gut, and corresponding increases in mucosal Treg numbers [30]. Indeed, with the growing body of literature supporting a role for inflammation in driving T2D [32], a clinical trial using experimental *N*. *americanus* infection is currently being conducted to investigate the therapeutic effect of helminth infections in metabolic disorders (Fig 2) [33]. Most of these early-phase clinical trials were impaired by the absence of a standardized manufacturing protocol for *N*. *americanus*. However, methods for the production of cGMP human hookworms were recently described [34] and are essential advances if helminth therapy is to receive widespread acceptance by the medical fraternity [35].

Further to therapeutic trials of helminth infections in inflammatory disease settings, dose-escalation controlled helminth infections in healthy volunteers are currently ongoing (S1 Table), primarily intending to develop a platform to test anti-helminth vaccines and drugs [34, 36].

## Insights into parasite–host interactions from animal models

### Excretory-secretory products

Helminths secrete bioactive molecules that can suppress or skew host immune responses; collectively, this suite of molecules is referred to as excretory/secretory products (ESPs). ESPs are a complex mixture of proteins, peptides, nucleic acids, lipids, glycans, and small organic molecules. Administration of ESPs from many nematodes and platyhelminth species induces immune responses that reflect the active infections. Moreover, ESPs have therapeutic properties in a range of animal models of autoimmunity, allergy, and metabolic disease (see recent reviews [37–39]). Indeed, the use of ESPs instead of active helminth infection potentially addresses some of the drawbacks and obstacles currently faced by experimental helminth therapy [38].

ESPs from many helminths, including at least 2 hookworm species (*Ancylostoma caninum* and *A*. *ceylanicum*), protected mice against T-cell-dependent trinitrobenzene sulfonic acid (TNBS)-induced colitis [39] and T-cell-independent dextran sulfate sodium (DSS) colitis [40, 41]. Prevention of immunopathology with ESPs is not only restricted to diseases driven by T_H_1/T_H_17 responses; both immuno-epidemiological observations [42] and mouse studies [43, 44] have shown that helminths and ESPs are also potent suppressors of T_H_2-driven allergic inflammation. The ability of ESPs to regulate all major forms of immunopathology is primarily attributed to its potent regulatory properties [45, 46]. Helminth ESPs drive a modified T_H_2 response that is different from the canonical T_H_2 response seen in allergies in which activated CD4^+^ cells secrete T_H_2 cytokines, including interleukin (IL)-4, IL-5, and IL-13 [47]. ESPs instead induce a modified T_H_2 response characterized by the secretion of regulatory cytokines such as IL-10 and the immunosuppressive transforming growth factor-beta (TGF-β) to ensure the containment of infection to manageable levels and that excessive T_H_2 inflammation does not ensue [48].

### ESPs target innate immune cell function

Parasitic helminths target several cell types that orchestrate a characteristic immune regulatory phenotype, notably, antigen-presenting cells such as DCs and macrophages. DCs possess intrinsic tolerance mechanisms that are integral initiators of the T_H_2 response [49]. Regulatory or tolerogenic DCs expressing integrin alpha E (CD103) are located in the intestinal mucosa and mesenteric lymph nodes [50]. Mucosal CD103^+^ DCs are capable of antagonizing T_H_2 induction in *Schistosoma mansoni* and *Heligmosomoides polygyrus* infections in mice. Mucosal CD103^+^ DCs secrete TGF-β, retinoic acid, and IL-2 and prevent immune-mediated pathology by promoting the expansion of Treg populations and maintaining intestinal homeostasis. Helminth ESPs target DCs and macrophage activation through suppression of pattern recognition receptor function, diminishing the ability of these innate sentinels to detect and respond to pathogen-associated molecular patterns (reviewed in [37]). Hookworm ESPs induce CD103^+^ DCs to express retinoic acid, which, in turn, facilitated the expansion of Tregs in a mouse model of asthma [51]. Moreover, helminth ESPs are known potent inducers of M2 macrophages [52], a cell population that drives T_H_2 responses and promotes wound repair [2].

Recent studies have shed light on the mechanisms by which helminths initiate T_H_2 responses by signaling through ILC2s (reviewed in [53]). ILC2s are enriched in the mucosa of gastrointestinal nematode–infected mice and arise in response to secretion of the alarmin IL-25 by intestinal tuft cells [54]. ILC2s respond rapidly to the presence of helminths by increasing in number and secretion of type 2 cytokines [54]. This cascade, in turn, results in the repair of epithelial barriers and recruitment of other innate cells, including eosinophils and culminates in the activation of T_H_2 cells and regulatory pathways.

### Helminths induce Treg activity

Tregs constitute around 5% of circulating CD4+ T cells and are identified by the lineage marker forkhead box P3 (FOXP3). Mutations in several genes that orchestrate Treg function, including the IL-2 receptor alpha subunit CD25 and cytotoxic T-lymphocyte-associated protein 4 (CTLA-4) result in the development of severe autoimmune syndromes [55], so Tregs constitute a significant target for new therapeutic strategies. Such research is focusing on the ability of Tregs to mediate immune regulation via multiple mechanisms, including IL-2 deprivation, secretion of the regulatory cytokines IL-10 and TGF-β, and acquisition of costimulatory molecules from antigen-presenting cells through binding to CTLA-4.

**Fig 3:**
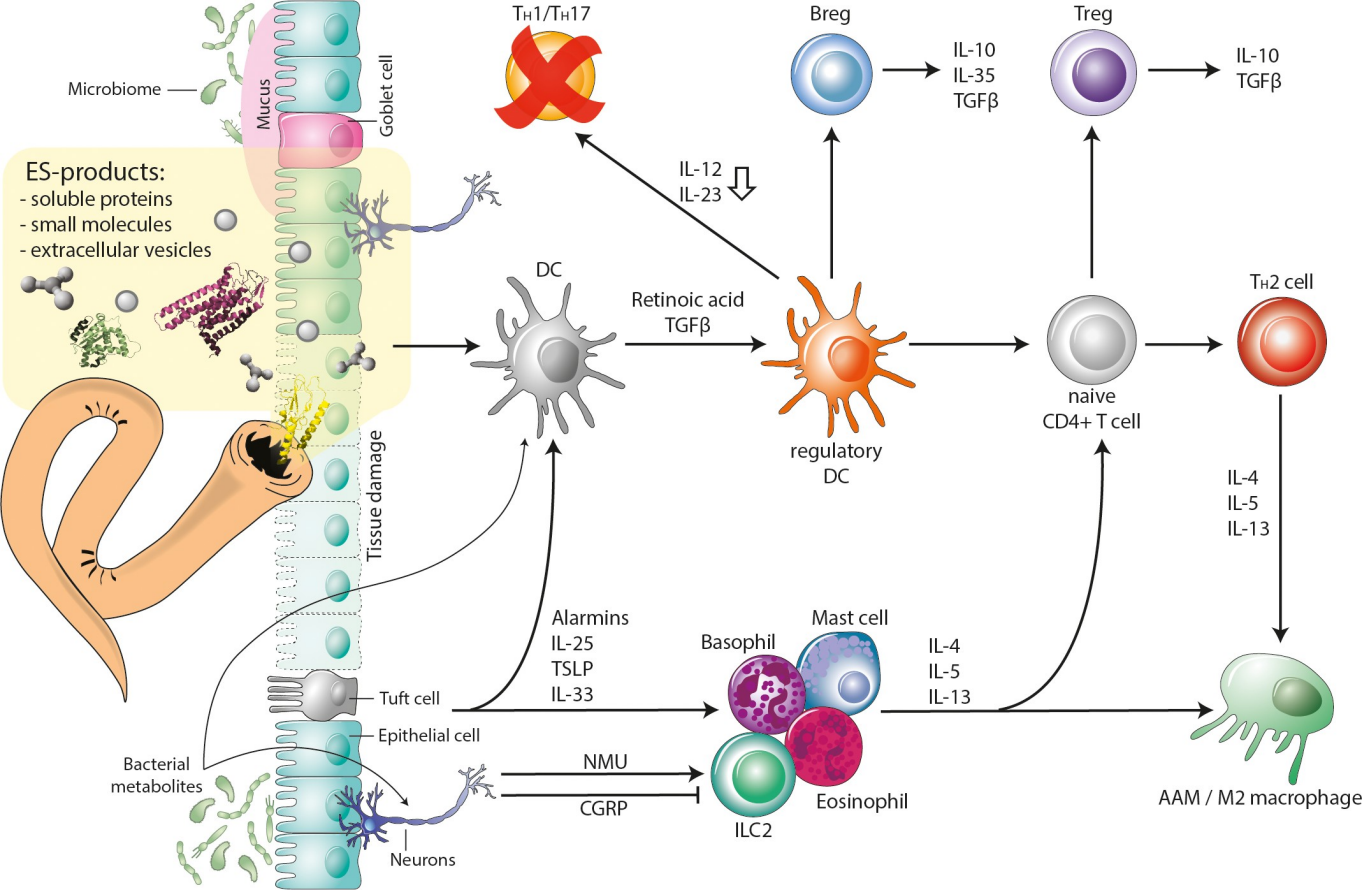
Helminths and their ESPs manipulate the host immune system. Helminth infection promotes T_*H*_2 cell differentiation, Treg responses, macrophage polarization, and mucus production, which are regulated by multiple upstream events and stimulated by signals from the worm (ESPs), but also signals from the microbiome (metabolites) and tissue damage (alarmins). DCs are central to these processes and respond to alarmins, ESPs, and metabolites to adopt a regulatory phenotype that promotes Treg, Breg, and T_*H*_2 cell development and suppress T_*H*_17 and T_*H*_1 cell responses. In addition, helminth-induced damage to the epithelium causes the release of alarmins such as TSLP, IL-25, and IL-33 from tuft cells and other epithelial cells, which can act on ILC2s and granulocytes to augment production of type 2 cytokines. This network is also influenced by sensory neurons within the gut that sense signals from helminths and microbes and elicit production of neuropeptides such as NMU and CGRP to regulate ILC2 responses directly. Breg, regulatory B cell; CGRP, calcitonin gene-related protein; DC, dendritic cell; ESPs, excretory/secretory product; ILC2, type 2 innate lymphoid cell; NMU, neuromedin U; T_*H*_2, T helper type 2, TLSP, thymic stromal lymphopoietin; Treg, regulatory T cell.

Both nematodes and platyhelminths are potent drivers of Treg responses. In mice infected with either *Nippostrongylus brasiliensis* or *S*. *mansoni*, IL-4 receptor alpha-mediated signaling on Tregs was required in vivo for control of helminth-induced inflammation [56]. *H*. *polygyrus* ESPs directly induces Tregs in vitro using FOXP3-green fluorescent protein reporter mice [49], and more recently, several helminth recombinant proteins have been shown to drive Treg expansion [37]. Indeed, from a clinical perspective, molecules that drive expansion, mobilization, or increased mucosal homing of 9Tregs are the holy grail for many disorders that result from immune dysregulation [57, 58], and we will provide defined examples later in this review. Regulatory B cells (Bregs) are also a feature of helminth infections [59] and are the primary source of IL-10 and other tissue-protective proteins, including (Resistin-like molecule-α) RELMα [60, 61]. Indeed, in mice infected with *H*. *polygyrus*, IL‐10^+^ Breg cells were able to promote expansion and maintenance of IL‐10^+^ FOXP3^+^ Treg cell populations [61]. Fig 3 summarizes the multitude of cellular immune pathways upon which helminths and their ESPs impact, including the well-established influence of DCs, but also the emerging potential roles for factors produced by microbes, sensory neurons, and specialized intestinal epithelial cells that initiate the very early response to helminth exposure via ILC2s and innate granulocytes [62, 63]. These recently identified biological pathways may, therefore, be potential therapeutic targets for immunoregulation by helminths and their secreted products.

### Helminth therapeutic moieties

Despite the wealth of literature supporting the therapeutic use of helminth ESPs for treating inflammatory disorders, the capacity of a crude parasite-derived supernatant to be developed as a drug is limited. ESP proteomes from many distinct species of helminths have been characterized, and their annotation has been supported by the increasing number of draft genomes [64]. Although a handful of individual proteins with immunoregulatory properties have been identified from ESPs, helminth-secreted proteomes still present a relatively untapped pharmacopeia [65, 66]. Next, we highlight a select group of molecules with proven immunoregulatory roles and potential therapeutic properties.

### Proteases and protease inhibitors

There are numerous examples of proteins from multicellular ecto- and endoparasites that have evolved from a protease or protease inhibitor scaffold but no longer possess canonical activity [65–67]. For example, 2 of the most abundant ESP proteins in *A*. *caninum* possess an ancestral netrin domain and are structural homologs of the tissue inhibitor of metalloprotease (TIMP) family [68]. Although *A*. *caninum* anti-inflammatory protein (*A*c-AIP)-1 and *Ac*-AIP-2 possess a TIMP-like domain, they do not appear to have the ability to suppress matrix metalloprotease catalytic activity and instead have evolved a distinct anti-inflammatory function [67, 69]. Recombinant AIPs suppressed expression of DC activation and costimulation markers [52, 70] and drove the subsequent expansion and mucosal homing of Tregs, which protected against inducible asthma [51]. Prophylactic treatment of mice with recombinant *Ac*-AIP-1 in chemically induced colitis protected mice against weight loss, clinical disease, and intestinal histopathology and significantly reduced the expression of hallmark T_H_1/T_H_2/ T_H_17 cytokines that drive inflammation in human IBD [71].

In similar fashion to the plasticity of the TIMP-like domain in hookworms, there is growing evidence that throughout their evolution, some cysteine protease inhibitor (cystatin) superfamily members have acquired novel roles that are independent of cysteine protease inhibition [72]. Filarial nematodes secrete cystatins that possess the canonical papain-like enzyme inhibitory activity but have also evolved a novel function that allows them to inhibit the catalytically distinct asparaginyl endopeptidase activity. This dual function assists in the inhibition of antigen processing in the major histocompatibility complex (MHC) class II pathway [73]. Cystatins from various helminth species suppress the secretion of inflammatory cytokines and promote IL-10 production by macrophages in particular [74, 75]. Cystatin from the filarial nematode *Acanthocheilonema viteae* suppressed inducible colitis and asthma in mice and displayed ex vivo bioactivity with human peripheral blood mononuclear cells from atopic patients with grass pollen allergy [76]. Moreover, oral delivery of *A*. *viteae* recombinant cystatin via continual dosing of transgenic *Lactococcus lactis* prevented the onset of colitis in pigs [76]. Subsequent studies have shown that cystatins from other helminths, both nematodes, and platyhelminths, can suppress inducible colitis [77–79].

### Cytokine mimics and cytokine-binding proteins

There is a growing body of literature on helminth ESPs that possess cytokine-like functions but not necessarily cytokine-like sequence homology or secondary structure. For example, *H*. *polygyrus* secretes a protein called *H*. *polygyrus* TGF-β mimic (*Hp*-TGM) that binds to the mammalian TGF-β complex and drives human and mouse Treg production but has no sequence homology to mammalian TGF-β. Instead, *Hp*-TGM is a member of the complement control protein (CCP) superfamily [80]. Recombinant *Hp*-TGM delayed allograft rejection in mice and increased Treg numbers in draining lymph nodes at the site of graft transplant, highlighting a potential use for this protein in a range of inflammatory settings. In like fashion, *N*. *americanus* activation-associated secreted protein (*Na*-ASP-2) secreted by *N*. *americanus* infective larvae is a member of the sperm-coating protein (SCP/TAPS) family and has structural- and charge-mimicking features of CXC-chemokines that recruit Tregs [81]. *Na*-ASP-2 was shown to bind to CD79A on human B cells whereupon it affected the expression of genes involved in leukocyte transendothelial migration [82].

*H*. *polygyrus* secretes a cytokine-binding protein called *H*. *polygyrus* alarmin release inhibitor (*Hp*ARI). This protein also belongs to the CCP family and inhibits the release of alarmins [83]. *Hp*ARI binds directly to IL-33 and nuclear DNA, thereby tethering the alarmin within necrotic cells and preventing its release. Recombinant *Hp*ARI administered to mice intranasally suppressed ILC2s and eosinophil responses in the lungs of mice after administration of *Alternaria* allergen, highlighting its utility for treating inflammatory diseases in the lungs in particular.

### Helminth molecules that accelerate wound healing

Helminths penetrate and migrate through skin and tissues without causing significant damage. The mechanisms employed by helminths to concurrently reduce injury and stimulate healing in the host are currently being explored [61, 84–86]. Most of the existing literature focuses on the role of the T_H_2 response—type 2 macrophages in particular—in driving wound repair; however, a handful of helminth-secreted molecules with wound healing properties have been described. The liver fluke, *Opisthorchis viverrini*, secretes a granulin (GRN)-like growth factor, *Ov*-GRN-1, which promotes wound healing and angiogenesis [87]. Recombinant *Ov*-GRN-1 is challenging to express in scalable form, so a readily-synthesized bioactive peptide fragment of *Ov*-GRN-1 that retained both in vitro and in vivo wound healing properties was identified and retained the in vivo therapeutic properties of the parent protein [88]. Given the alarming incidence of T2D and associated comorbidities such as nonhealing diabetic foot ulcers, topical growth factor-like proteins and peptides from helminths address an area of great unmet need [89, 90].

### Post-translational modifications

Many helminth immunomodulatory proteins are secreted and therefore undergo post-translational modifications (PTM). In some cases, the PTM are responsible for the addition of the regulatory moiety of interest. For example, the dominant ESP of *A*. *vitae* is ES-62. This glycoprotein that has therapeutic effects in a range of mouse models of inflammatory disorders such as arthritis [91], asthma [92], and even systemic lupus erythematosus [93]. ES-62 is an aminopeptidase that carries *N*-glycans decorated with phosphorylcholine (PC) [94, 95]. The fusion of PC to an unrelated carrier protein demonstrated that it retained its therapeutic properties, thus proving that PC is the bioactive moiety of ES-62 [96]. Small drug-like analogs of PC for the treatment of arthritis and chronic lung fibrosis have overcome immunogenicity concerns with the sizeable ES-62 protein [97, 98].

Chemical deglycosylation of ESPs from some helminths ablates protection against inflammatory diseases [99], highlighting the importance of glycans in driving regulatory responses. One such glycan that decorates schistosome soluble egg antigens (SEAs) and secreted egg proteins is the Lewis X-containing glycan found on dominant egg proteins such as omega-1 and interleukin-4-inducing principle (IPSE)/alpha-1 [100]. Recombinant IPSE/alpha-1 expressed in wild tobacco drives IL-10 production from Bregs [101] and suppresses inflammatory cytokine responses by skewing inflammatory monocytes toward anti-inflammatory M2 macrophages [102]. Administration of SEA to mice induced T_H_2 immune responses characterized by M2 macrophages and eosinophils in WAT and liver and reduced fat mass gain and lowered insulin resistance and glucose intolerance [103, 104] (Fig 2). Protection against metabolic disease was dependent on the engagement of the mannose receptor CD206 and the release of IL-33, inducing ILC2-dependent improvements in metabolic status [105]. The immunomodulatory Lewis X-containing glycan, lacto-N-fucopentaose III (LNFPIII) from the schistosome tegument also improves glucose tolerance and insulin sensitivity in diet-induced obese mice, at least in part via increased IL-10 production by macrophages and DCs, and resulted in reduced WAT inflammation [106]. LNFPIII treatment also up-regulated the expression of the multipurpose farnesoid X nuclear receptor (FXR)-α to reduce lipogenesis in the liver, thereby protecting against hepatosteatosis [107]. Producing appropriately glycosylated recombinant versions of schistosome glycoproteins has proven challenging. Nevertheless, recent efforts to glycoengineer Lewis X-containing proteins in wild tobacco with coexpression of defined glycosyltransferases proved successful [105] and opened up new possibilities for generating appropriately glycosylated immunotherapeutics.

### Helminth metabolites—an untapped resource for small-molecule therapeutics

Although helminth proteins and their PTMs have been documented and discussed elsewhere in this review, much less is known about parasitic helminth metabolomes. The metabolomics revolution has begun to reveal small-molecule metabolites in parasitic helminth somatic extracts and ESPs [108–111]. Helminthology has been slow to adopt cutting edge metabolomics techniques [111], partly because of the difficulty in obtaining sufficient quantities of ESPs, but also because of the diversity in physicochemical properties of metabolites results in it being almost impossible for a single analytical method to provide the required full coverage of the metabolome of a given biological sample. As such, particular analyses are biased toward certain groups of metabolites. Only a handful of studies have characterized helminth metabolomes, and even fewer have addressed the anti-inflammatory properties of these molecules. *A*. *caninum* secretes metabolites that suppress both inducible colitis in mice and ex vivo production of inflammatory cytokines from human PBMCs [112]. The identity of the protective moieties is not yet known, but short-chain fatty acids secreted by the parasite (or the hookworm’s resident microbiota) are prospective candidates. Another candidate is succinate, a metabolite that is produced by the intestinal microbiota that is critical for inducing intestinal tuft cells to initiate T_H_2 responses. Succinate was recently shown to be a major component of metabolomes of gastrointestinal helminths [109, 112] and was significantly enriched in the ESP metabolome of hookworms compared to the somatic tissue metabolites.

The lipidome of different developmental stages of *S*. *mansoni* was recently described, and the egg stage was shown to be enriched in oxylipids with known immunoregulatory properties such as prostaglandins [113]. Indeed, prostaglandin E2 is secreted in high amounts by *T*. *suis* compared to other lipids and suppresses TNF and IL-12 secretion from lipopolysaccharide-activated human DCs [114]. Of course, prostaglandins are not unique to helminths and are a therapeutic target in their own right because of their up-regulation in some aggressive cancers [115]. Helminth metabolites that are likely to be candidate small-molecule drugs are those that are unique to parasites and have evolved to suppress defined immunopathological pathways safely and are readily synthesizable. We anticipate substantial growth in this area in the coming years given the increased access of researchers from diverse fields to metabolomics platforms.

### Helminth-secreted extracellular vesicles in the treatment of inflammatory disorders

The discovery that parasitic helminths secrete extracellular vesicles (EVs) has spurred a new paradigm in the discovery of helminth-derived immunotherapeutics and antihelminth vaccines [116, 117]. EVs are a heterogeneous group of lipid-enclosed vesicles in the nano- to the micrometer size range. There is increasing evidence that helminth EVs are essential players in regulating host inflammation and immunity, and their application as anti-inflammatory therapeutics has been considered. However, to date, the exact mechanisms by which helminth EVs polarize immune responses remain elusive.

In the *Alternaria* model of allergic asthma, administration of *H*. *polygyrus* EVs significantly reduced lung immunopathology via ILC2-mediated suppression of innate immunity [118]. The anticolitic therapeutic potential of helminth EVs has also been demonstrated in several recent reports. *N*. *brasiliensis* EV-treated mice were protected from T-cell-dependent acute colitis, specifically by the suppression of inflammatory cytokines and increased expression of IL-10 [119]. EVs from the liver fluke *Fasciola hepatica* can modulate T-cell-independent colitis [120], characterized by reduced expression of intestinal proinflammatory cytokines that suppress both mitogen-activated protein kinase (MAPK) and nuclear factor kappa-light-chain-enhancer (NF-κB) signaling pathways.

Perhaps the most intriguing aspect of helminth EVs (from a drug development perspective) is the abundance of vesicular microRNAs (miRNAs) that are predicted to target mammalian host genes, particularly those involved in immune processes. Helminth EVs are actively internalized by host cells [119, 121], providing a mechanism by which the parasites transfer genetic material to the host in a bid to actively manipulate host gene expression [116, 118]. There is a growing body of literature demonstrating the presence of parasite-specific miRNAs secreted by worms, but little is known about their specific interactions with host genes. *H*. *polygyrus* miRNAs were shown to down-regulate the expression of the mouse phosphatase gene *dusp1* (which corresponds to MKP-1 in humans), a key regulator of MAPK signaling and T_H_1 responses to toll-like receptor ligands using a luciferase reporter assay [118].

Looking forward, a deeper understanding of helminth miRNA immunobiology may lead to advances in miRNA-based therapeutics for a whole host of immune dysfunction diseases, including conditions like scleroderma, in which defined miRNAs (of host origin) are already in preclinical and clinical development (reviewed in [122]). EV research has been critical for demonstrating how miRNAs could be used to treat disease, but some practical challenges and obstacles need to be overcome before this type of therapy enjoys mainstream application [123].

### Turning worm proteins into conventional drugs

The molecular diversity of known helminth ESPs with therapeutic properties is impressive, and considering that we have only dipped our toes in the water, the potential breadth of moieties is prodigious. Each family of molecules and even individual members within those families present unique and shared challenges. Moreover, the pharmacokinetic (PK) properties of a helminth-derived therapeutic protein and how best to deliver it to the target tissue/organ are challenging. For example, an ideal IBD drug would be delivered orally, and although some helminth ESPs have likely evolved to be stable in acidic environments, ensuring that a protein enjoys safe passage through the stomach and delivery to the specific cell type in the gut is not straightforward. Moreover, what does the optimal PK profile of a helminth protein look like? Unlike most biologics (e.g., monoclonal antibodies) in which plasma half-life can be measured in weeks, a helminth protein might only be present for hours. This longer half-life might, however, be sufficient for enduring functional activity via impact on defined cell types and mobilization of those cells to inflamed tissues [51]. The foreignness of a helminth protein also poses potential immunogenicity concerns, and the development of antidrug antibodies could be problematic.

Furthermore, the recombinant expression platform is of paramount importance for the production of helminth biologics. Many groups are expressing recombinant helminth proteins in cell lines that are used for industrial-scale production of antibodies and other therapeutic proteins. Levels of endotoxin and other host cell-derived contaminants need to be strictly controlled for cGMP grade production of biologics, particularly for immunotherapeutics that aim to wind down inflammatory responses (as opposed to promoting them with vaccines), so an emphasis on eukaryotic cell expression platforms, such as HEK293, is recommended.

Helminth-derived miRNAs are predicted to target an impressive array of mammalian host genes involved in inflammation, but how those miRNAs might be therapeutically delivered to target cells, internalized, and then encounter their target gene is not clear [124, 125]. In the natural setting, many miRNAs are delivered to target cells in the sheltered environment of EVs, but recapitulating this process for a therapeutic purpose requires novel approaches, such as the use of synthetic exosomes [126].

Despite these challenges, evolutionary selection pressure has tailored helminth ESPs to be efficacious and safe, at least in the setting of active helminth infection. Of course, in a therapeutic setting, the dose, the frequency, and route of administration are likely to be different. Furthermore, it is important to note that numerous nature-inspired drugs, sourced from the venom of various invertebrates and vertebrates, are commercially available for treating a range of disorders [127]. It is timely that helminths now join this list and that drugs inspired by these exquisitely adapted parasites get the attention they deserve. The scientific community implores industry-based organizations to make long-term investments intended at deciphering and capitalizing on the extraordinary and diverse modes of action of these products to unearth the next generation of novel therapeutics.

## Supporting information

S1 Table Completed and ongoing experimental helminth infection studies in healthy volunteers. (DOCX)
