# Supplementary material for: Harnessing helminth-driven immunoregulation in the search for novel therapeutic modalities
Source: PLoS Pathog. 2020 May 14;16(5):e1008508. doi: 10.1371/journal.ppat.1008508 (PMC7224462; doi:10.1371/journal.ppat.1008508)
Supplement: S1 Table — (DOCX) [file ppat.1008508.s001.docx]

Supplemental Table 1: Completed and On-going Experimental Helminth Infection Studies in Healthy Volunteers.

| Trial / phase | Helminth | Status  (year range) | Study Title and treatment | Outcome | Reference |
| --- | --- | --- | --- | --- | --- |
| NCT01940757  Phase 1 | *N. americanus* larvae | 2015 - 2021  (recruiting) | **Hookworm Vaccination-Challenge Model (HVCM)**  Non-randomized, open-label, dose-escalation clinical study in healthy, hookworm-naïve adults (n=30). | Consistent induction of patent *N. americanus* infection suitable for future HVCM trials was achieved with the inoculum of 50 NaL3. | [34] |
| NCT03126552 | *N. americanus* larvae | 2017 – 2020  (complete) | **Controlled human hookworm infection model (CCHI)**  Open-label single group assignment. Inoculated 50 *N. americanus* L3 larvae (n=4) followed for 52 weeks. | Well tolerated patent hookworm infection in all subjects and resulted in high egg counts. No serious adverse events. | [36] |
| NCT03257072 | *N. americanus* larvae | 2017 – 2018  (completed) | **Repeated Controlled Human Hookworm Infection (ReCHHI1)**  Randomized, double-blind, placebo-controlled, dose-escalation trial in healthy volunteers (n=24). Group A infected with 50 L3 at week 4 only, group B at week 2 and 4, while group C received L3 at weeks 0, 2 and 4. Group A and B received mock injections at week 0 and 2 to maintain blinding. 16 weeks after last injection subjects could choose albendazole treatment. | Unknown results | Leiden University Medical Center |
| NCT03702530 | *N. americanus* larvae | 2018 – 2019  (completed) | **Immunisation, Treatment and Controlled Human Hookworm Infection (ITCHHI)**  Randomized into either intervention or placebo group (2:1) allocation. Inoculated with 50 L3 larvae three times, followed by treatment with albendazole. | Unknown results | Leiden University Medical Center |
| ACTRN12619001129178 | *N. americanus* larvae | 2019 – 2020  (recruiting) | **Therapeutic Hookworm Phenotyping Study (CHHIM Study)**  Establishing a controlled human hookworm infection by application of 15 L3 larvae. No control group. | Unknown results | Malagan Institute of Medical Research, New Zealand |
| ACTRN12617001007325 | *N. americanus* irradiated larvae (irL3) | 2017 – 2018  (complete) | **Vaccination**  Randomized, placebo-controlled, safety and tolerability of a dose escalation (50 to 100) of attenuated (irradiated) hookworm larvae in healthy volunteers (cohort 1: 50 irL3 n=2, cohort 2: 100 irL3 n=2). Stage 1: 8 days following inoculation all participants will receive albendazole.  Stage 2: pilot randomised control trial 15 participants assigned 1:2 receive 2 doses of either placebo or challenge infection with irL3. 6-weeks post infection all participants to receive 30 normal hookworm larvae. All participants will receive albendazole at week 11 termination of study. | Unknown results | QIMR Berghofer, Australia |
|  |  |  |  |  |  |
| NCT02755324 | *Schistosoma mansoni* male cercariae | 2016 – 2019  (completed) | **Controlled *Schistosoma mansoni* infection model**  Open-labelled, single-sex (male *S. mansoni* cercariae) controlled human schistosomiasis infection: dose-escalating clinical safety findings (n=17). | Dose-related increase in adverse events. Serious adverse events observed in 18% of volunteers by infection with 20 *S. mansoni* cercariae. | [136] |
| NCT04269915 | *S. mansoni* female cercariae | 2020 – 2022 (on-going) | **Controlled Female-only *Schistosoma mansoni* infection model**  Open-labelled, safety and dose finding study (CoHSI2)  volunteers were exposed to escalating doses of female *Schistosoma mansoni* cercariae (n=22). | On-going | Leiden University Medical Center |
